# Risk factors and population attributable fraction for large-for-gestational-age and macrosomic births in low- and middle-income countries between 2000 and 2025: a protocol for systematic review and meta-analysis

**DOI:** 10.1136/bmjopen-2025-110407

**Published:** 2026-05-08

**Authors:** Fati Kirakoya-Samadoulougou, Hannah Blencowe, Dieudonné Ilboudo, Joyeuse Ukwishaka, Lorena Suarez Idueta, Elizabeth A Hazel, Eric Ohuma, Daniel J Erchick, Joanne Katz, Anne CC Lee, Robert E Black

**Affiliations:** 1 Centre de Recherche en Epidémiologie, Biostatistique et Recherche Clinique, Université libre de Bruxelles, Brussels, Belgium; 2 Department of International Health, Johns Hopkins Bloomberg School of Public Health, Baltimore, Maryland, USA; 3 Department of Infectious Disease Epidemiology and International Health, Faculty of Epidemiology and Population Health, LSHTM, London, UK; 4 Centre Muraz, Institut National de Santé Publique, Bobo-Dioulasso, Ouagadougou, Centre Region, Burkina Faso; 5 Warren Alpert Medical School and Global Alliance for Infant and Maternal Health Research, Brown University, Providence, Rhode Island, USA

**Keywords:** Risk Factors, Meta-Analysis, Mothers, Community child health

## Abstract

**Introduction:**

Large-for-gestational-age (LGA) and macrosomic births pose significant maternal and neonatal health risks, particularly in low- and middle-income countries (LMICs), where access to care are often limited. Despite well-established associations between LGA, macrosomia, and various risk factors, the relative contributions of these factors remain underexplored in LMICs. This study aims to identify risks factors for LGA and macrosomia in LMICs, with an emphasis on modifiable ones, and quantify their population attributable fractions (PAFs).

**Methods and analysis:**

A systematic review will be conducted across the following databases: MEDLINE, Scopus and ProQuest Central and regional databases (Africa Index Medicus, Index Medicus for South Asia and Latin America and Caribbean literature of health sciences). Eligible studies will include observational studies, reviews and interventional research conducted between 2000 and 2025 that report on prevalence or association of risk factors for large-for-gestational-age (LGA) and/or macrosomia births in low- and middle-income countries (LMICs). Data extraction will encompass study characteristics, prevalence/incidence estimates, risk factor distributions and measures of association. Quality assessment will be performed by two independent reviewers using the Newcastle-Ottawa Scale for observational cohort, case–control and cross-sectional studies. While Cochrane Risk of Bias Tool will be used for randomised controlled trials and a Measurement Tool to Assess Quality of Systematic Reviews 2 (AMSTAR-2) for systematic reviews and meta-analyses. Meta-analyses using a random-effects model, which accounts for population heterogeneity, will synthesise risk estimates for factors examined in three or more studies from LMICs, up-to-date meta-analysis including all relevant studies identified through our search. Population attributable fractions for individual and combined risk factors will be calculated.

**Ethics and dissemination:**

This systematic review will use only previously published information. Ethical approval is therefore not required. The results will be submitted for publication in a peer-reviewed journal and the findings will be presented at international conferences to engage relevant stakeholders including policymakers and public health organisations in LMICs with the aim of informing the development of targeted interventions to reduce the burden of LGA and macrosomia births in the region.

**STRENGTHS AND LIMITATIONS OF THIS STUDY**

- The study will use a systematic search strategy, including multiple databases to ensure thorough coverage of the topic.
- The study will use appropriate tools to assess the quality and risk of bias of the included studies, with two independent reviewers ensuring the quality of the studies.
- This study will help determine the contribution of key risk factors to the occurrence of large-for-gestational-age (LGA)/macrosomia and inform decision-makers on high-impact interventions to manage LGA/macrosomia risk, particularly in high-risk populations in low- and middle-income countries.
- The use of pooled or regional prevalence estimates when country-level data are unavailable may limit contextual specificity and the generalisability of findings.
- Converting ORs to relative risks for population attributable fraction calculation may introduce some imprecision, particularly when it relies on external or assumed baseline outcome prevalence.

## Background

 Large-for-gestational-age (LGA) and macrosomia represent significant public health concerns, affecting up to 22% of births worldwide.^1^,^3^ Compared with appropriate-for-gestational-age or normal-weight newborns, LGA and macrosomia are associated with elevated risks of birth trauma, neonatal hypoglycaemia, respiratory distress and later-life metabolic disorders.^2 4 5^ These risks are particularly pronounced in low- and middle-income countries (LMICs), where limited access to emergency obstetrical and neonatal care compounds adverse outcomes.^6 7^ Multicountry analyses confirm that macrosomia increases the likelihood of caesarean delivery and adverse maternal outcomes globally, with the strongest neonatal effects observed in Africa.^2^ Despite these implications, evidence on the risk factors for LGA and macrosomia in LMICs remains limited.

Multiple maternal, fetal and environmental factors contribute to excessive fetal growth. These include gestational diabetes, maternal obesity, multiparity, history of LGA/macrosomia, excessive gestational weight gain and male fetal sex.^28^,^15^ Additional determinants such as advanced maternal age, nutrition, socioeconomic status, tobacco or alcohol use, stress and genetic predispositions further increase risk.^16 17^ Among these, gestational diabetes, maternal body weight and maternal lifestyle before and during pregnancy are the most consistently modifiable risk factors.^810 16 18^,^21^ Interventions addressing these factors particularly through early detection and management of gestational diabetes and improved maternal health behaviours have shown promise.^22^,^25^ However, the implementation of such strategies in LMICs has been limited, despite rising prevalence of LGA and macrosomia and the urgency to reduce maternal and neonatal mortality in line with Sustainable Development Goal 3.2.^26^

In LMICs, data on the relative contribution of these risk factors remain sparse. Past limitations included incomplete reporting of birth weight and gestational age, as well as heterogeneity in diagnostic methods across health systems, complicating the estimation of population attributable fractions (PAFs).^27^,^29^ Recent improvements in routine data collection have now enabled more robust analyses. Understanding which risk factors drive LGA and macrosomia, and quantifying their population-level impact, is critical for developing cost-effective and context-specific interventions.

This systematic review and meta-analysis aim to identify risk factors for LGA and macrosomia in LMICs, with an emphasis on modifiable ones and quantify their population attributable fractions. By identifying these contributing factors, the study seeks to inform targeted strategies to mitigate LGA and macrosomia-related health risks in LMICs.

### Conceptual framework

**Figure 1:**
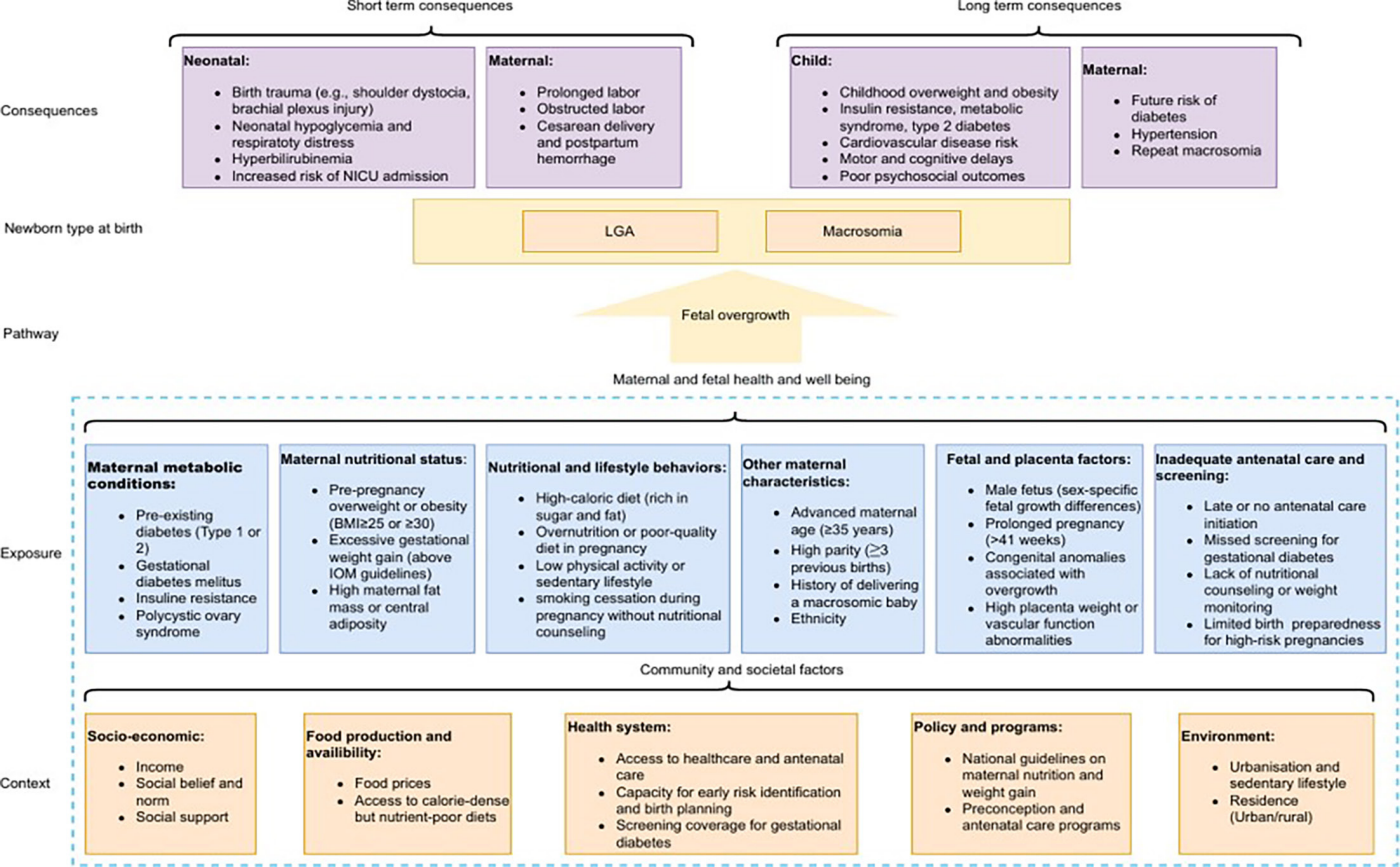
Conceptual framework illustrating the interplay of factors leading to LGA and macrosomia and their influence on early and late childhood outcomes, as informed by previous studies.^25 8 10^,^16 19 26 30^ BMI, body mass index; LGA, large-for-gestational-age; NICU, Neonatal intensive care units; IOM, Institute of medecine.

Based on an initial analysis of the literature we developed an initial conceptual framework for risk factors for LGA or macrosomia (in the blue box), as well as the potential implications in terms of maternal and neonatal complications (Figure 1).^25 8 10^,^16 19 26 30^ Following the conceptual framework, we identified the key risk factors associated with the occurrence of LGA or macrosomia (online supplemental table S1).

### Research question

This study addresses two primary research questions: (1) What are the established risk factors associated with LGA and macrosomia in LMICs? and (2) What proportion of LGA and macrosomia cases can be attributed to key risk factors?

## Methods and analysis

### Study design

This study will use a systematic review and meta-analysis to identify and quantify the association of key pre-pregnancy and during pregnancy risk factors with LGA and macrosomia births in LMICs. Where applicable, we will estimate the PAFs of these risk factors to assess their relative contribution to the burden of LGA and macrosomia. This protocol adheres to the Preferred Reporting Items for Systematic Reviews and Meta-Analyses Protocols (PRISMA-P) guidelines.^33^

### Information sources and search strategy

The search will be conducted across three major databases (MEDLINE, Scopus and ProQuest Central). We will also explore regional databases such as: Africa Index Medicus, Index Medicus for South Asia and Latin America and Caribbean literature of health sciences. The search strategy has been developed in collaboration with an expert librarian, based on Population, Concepts and Context framework, and combines Medical Subject Headings with free-text keywords, using Boolean operators (AND, OR). A pilot search was conducted on MEDLINE (PubMed) to test the effectiveness of the keywords and strategy. Based on the results, the tested search strategy will be refined and executed across the other selected databases. Additionally, reference lists from relevant studies will be manually reviewed to identify any additional sources. Electronic databases will be searched for studies published from January 2000 to December 2025, given the relevance of contemporary studies to research questions. The systematic review will be limited to studies published in English, French and Spanish, reflecting the language proficiency of the research team. This approach will allow direct, expert assessment of primary data, ensuring accurate interpretation of technical epidemiological information and maintaining internal validity, while also covering a substantial proportion of the scientific literature indexed in major international databases relevant to this topic. The final search will be supervised by an academic librarian to ensure the accuracy and completeness of the search process. The search strategy is provided in supplementary materials (online supplemental tables S1,S2 and S3).

### Study selection and eligibility criteria

This review will include observational studies and interventional trials conducted in LMICs.^34^ Systematic review, umbrella review and meta-analysis on this issue will be examined to verify the completeness of our search and to identify primary studies that may not have been identified through our search. Eligible studies must report on LGA births, defined as birth weight above the 90th or 97th percentile for gestational age, or macrosomia births, defined as birth weight ≥4000 g or ≥4500 g. Additionally, studies must also identify pre-pregnancy or during pregnancy risk factors associated with LGA or macrosomia. All studies reporting information on risk factors for LGA or macrosomia will be included in the systematic review. However, only studies providing quantitative data on the prevalence or relationships between risk factors and LGA or macrosomia will be included in the meta-analysis. Studies will be included irrespective of their size-for-gestational age assessment standard used (eg, INTERGROWTH-21st, WHO standards or locally derived).^35^,^37^ Studies will be excluded if they lack a clear definition of LGA or macrosomia, do not report primary data (editorials or commentaries), or do not provide data on prevalence or risk estimates (case reports).

### Study screening

The screening process will begin with the importation of identified articles into reference management software (EndNote), where duplicates will be removed. Following this, the remaining articles will be uploaded into Rayyan, a systematic review tool for efficient screening. Two independent reviewers (DI and JU) will then screen titles and abstracts from relevance based on the established inclusion and exclusion criteria. Full text articles will then be reviewed for final eligibility. In case of discrepancies between the reviewers, conflicts will be resolved through discussion or by consulting a third reviewer (FK-S).

### Quality of included studies

The quality of included studies will be assessed by two independent reviewers (DI and JU) using appropriate tools based on study design. For observational studies, we will use Newcastle-Ottawa Scale. Cochrane Risk of Bias Tool will be used for randomised controlled trials. Studies will be rated as having low, moderate or high quality. We will subsequently perform subgroup analyses to explore whether study quality modifies the effect estimates, by comparing results from studies rated as high quality to those rated as low quality. Any discrepancies in the assessment will be resolved through discussion or by involving a third reviewer.

### Selections of risk factors and estimation of measures of association

In the first step, we will identify and quantify associations between all risk factors and the outcomes of LGA and macrosomia in LMICs based on systematic review and meta-analysis. Only risk factors that have been examined in three or more eligible studies conducted in LMICs will be included in the meta-analyses, to pool ORs or relative risk (RRs) as previously done.^38^ For risk factors that are reported in fewer than three studies from LMICs, meta-analyses will not be conducted due to limited data and concerns over statistical reliability.^38^ Instead, these factors will be described in systematic review to highlight their potential contribution to LGA or macrosomia and to identify areas where further research is warranted.

A random-effects model will be used to account for potential variability across populations studied. Heterogeneity among included studies will be assessed using the *I*² statistic, to quantify the proportion of variation attributable to heterogeneity rather than random error. Publication bias will be evaluated through visual inspection of a funnel plot and statistical testing using Egger’s regression test.

In the second step, we will select the key risk factors based on their biological plausibility, the frequency with which they are reported in studies conducted in LMICs and the availability of interventions based on pre-conception and antenatal care.^39 40^ These risk factors will be organised into two categories: (1) pre-pregnancy factors (eg, maternal age, parity, birth interval, pre-pregnancy body mass index (BMI), pre-pregnancy physical activity) and (2) pregnancy factors (eg, antenatal care follow-up, gestational diabetes, weight gain during pregnancy, pregnancy physical activity, overnutrition or hypercaloric diet during pregnancy, post-term gestation) (online supplemental table S4).

### Prevalence of risk factors

For pre-gestational risk factors, we will estimate the prevalence of each risk factor using recent national household surveys (eg, Demographic and Health Survey or Multiple Indicator Cluster Survey) where available from WHO health inequalities monitor.^41^,^43^ Then, the regional average of these prevalences will be used for countries without nationally representative survey data.^44^ The prevalence of gestational risk factors, which are not measured in most national surveys, will be extracted directly from the primary studies included in the systematic review. Where such data are unavailable, the prevalence will be estimated by considering those produced by the Institute for Health Metrics and Evaluation (IHME) or meta-analysis that provide regional estimates of these risk factors.^20 45^ Finally, the weighted prevalence of each risk factor by WHO region will be determined.^44^

The sources used to estimate the prevalence of risk factors will be classified into three quality categories. ‘High’ quality sources correspond to prevalences derived from nationally representative surveys.^44^ ‘Medium’ quality sources include prevalences extracted directly from the primary studies included in this systematic review or estimated by the IHME. ‘Low’ quality sources comprise regional estimates obtained from literature reviews and applied at the national level, or estimates that are not specific to pregnant women or women of reproductive age.^44^

Based on a review of the literature, we found that certain risk factors (gestational diabetes and pre-existing diabetes in pregnancy) were the subject of intervention during pregnancy in these countries. The prevalence of gestational diabetes and pre-existing diabetes will be adjusted to account for the effect of treatment that prevents the effect of the risk factors, using the following equation:^46^

Adjusted prevalence=(unadjusted prevalence×coverage of treatment×efficacy of treatment).

We will estimate treatment coverage by multiplying the estimated coverage of at least four antenatal care visit (ANC4+) by an estimate of ANC quality (specifically, the percentage of facilities willing to screen and manage diabetes).^44^ Details of the potential data sources that will be used to extract risk factor prevalences can be found in online supplemental table S2.

### Estimation of LGA/macrosomia prevalence to determine number of LGA/macrosomia births

Our primary sources will be systematic review and meta-analysis on the prevalence of LGA and macrosomia in 23 LMICs conducted by Kirakoya-Samadoulougou *et al*.^26^ For the countries included in the meta-analysis, we will consider the national prevalence provided by this study. For countries not included in this meta-analysis or where national prevalence data are not available, we will consider the regional estimates of LGA and macrosomia prevalence in sub-Saharan Africa, South Asia, Latin America and Caribbean and East, Southeast and Oceania provided by the meta-analysis.^26^ We will consider all standard definitions of LGA and macrosomia (LGA as birth weight >90th or >97th percentile for gestational age and sex, and macrosomia as birth weight ≥4000 g or ≥4500 g). In countries lacking LGA data, we will use only macrosomia estimates if available.

### Estimation of live births

We will obtain country-specific projections of live births from the 2024 revision of the United Nations World Population Prospects, which provides standardised estimates of population size, fertility and annual live births for all United Nations member states.^47^ To estimate the total number of LGA or macrosomia births per country, we will multiply the country-specific prevalence of LGA or macrosomia (as described above) by the projected number of live births.

Estimated LGA/macrosomia births=prevalence of LGA/macrosomia×number of live births

This process will be applied to all countries included in the list of LMICs proposed by the World Bank Group.^34^ The resulting estimates will enable us to quantify the absolute burden of LGA or macrosomia births by country.

### Data extraction

Data will be extracted by two independent reviewers using a standardised data extraction form to collect essential study information. This will include study characteristics such as authors, year of publication, country or region, study design and sample size. Outcome data will focus on the prevalence of LGA and macrosomia, with LGA defined using both >90th and >97th percentile thresholds, and macrosomia defined as birth weight ≥4000 g or ≥4500 g, along with birth weight and gestational age. We will also extract information on the specific growth standard or reference used in each study, as well as the gestational age assessment method (ultrasound vs Last Menstrual Period, LMP) and the exact definitions of LGA and macrosomia applied. Additional data on maternal (eg, BMI, gestational diabetes, pre-existing diabetes), environmental (eg, tobacco use) and systemic (eg, access to prenatal care) risk factors will be recorded, along with their associated effect sizes, such as ORs or RRs. In case where PAFs are reported in the included studies, their estimates and calculation methods will be documented.

### Data synthesis and analysis

The study characteristics (study design, publication year and country or region), methodological approaches (study population, sample size and exposure definitions) and key findings (eg, prevalence of each risk factor, RRs or ORs and prevalence of LGA/macrosomia, where available) will be summarised to provide a comprehensive overview of the existing evidence.

After identifying RRs or ORs for each risk factor of LGA/macrosomia from the included studies, ORs will be converted to RRs using the prevalence of LGA/macrosomia in the reference group following equation 1.^48^ If this prevalence is not reported, we will use the most recent national prevalence; if unavailable, the overall regional prevalence (Africa, Asia or Latin America) will be used.^26^

Equation_1: $RR=\frac{OR}{\left(1-Po\right)+(Po\times OR)}$

Where OR: OR and Po= prevalence of LGA and macrosomia in the reference group.

Building on these estimates, the country-specific PAF for each risk factor will be calculated using the corresponding RR and the national prevalence of that risk factor, following the standard PAF formula.^49^

Equation_2: $PAF=\frac{Pe(RR-1)}{Pe\left(RR-1\right)+1}$

We will then estimate the country-specific combined PAF for all risk factors and the adjusted PAF for each risk factor, based on the individual and combined PAFs, respectively.

Equation_3: $CombinedPAF=1-\prod_{i=1}^{k}\left(1-PAFi\right)$

Where PAFi: population-attributable fraction for each individual risk factor i.

K: total number of risk factors.

∏: Product symbol (multiplying terms together).

Equation_4: $PAFa=\frac{IndividualPAF\mu }{\left(\sum PAF\mu 1-k\right)}\times PAFt$

Where k is the number of identified risk factors and PAFt is the combined PAF from equation 3.

Finally, using the adjusted PAFs, we will estimate the number of LGA/macrosomia births in each country attributable to each risk factor. We will then calculate the total number of LGA/macrosomia cases attributable to all risk factors and to modifiable risk factors at the country, and regional level.

In subgroup analyses, we will compare common thresholds for LGA (90th vs 97th percentile) and macrosomia (≥4000 g vs ≥4500 g). We will also compare studies using INTERGROWTH versus other growth references and conduct stratified analyses by gestational age assessment method (ultrasound vs LMP), where reported. While these stratifications help account for methodological differences, residual confounding may persist due to within-subgroup variability in gestational age assessment, growth references and outcome definitions, as well as heterogeneity in measurement accuracy across settings. This variability may slightly affect the comparability of effect estimates and attributable fractions.

To account for changes in the epidemiological and nutritional context in LMICs over time, we will conduct a temporal sensitivity analysis. Pooled risk factor estimates will be compared across two periods (2000–2012 and 2013–2025). This division reflects the introduction of the WHO’s 2013 diagnostic criteria for gestational diabetes and the growing burden of maternal metabolic disorders in LMICs,^50 51^ both of which may influence the risk profile for LGA/macrosomia.

### Scope of PAF estimation

This study will limit the estimation of PAFs to all countries included in the list of LMICs proposed by the World Bank Group.^34^

#### PAF calculation for regional and overall estimation

To quantify the contribution of individual and combined risk factors to LGA and macrosomia, we will calculate the PAFs using established models.

Equation_5: Regional and overall combined PAF.


$$PAF_{regional}=\frac{\sum_{i=1}^{n}\left(PAFci\times Nci\right)}{\sum_{i=1}^{n}Nci}$$

PAFci: PAF in country i.

Nci: Number of live births in country i.


$$PAF_{overall}=\frac{\sum_{i=1}^{n}\left(PAFi\times Nri\right)}{\sum_{i=1}^{n}Nri}$$

PAFri: PAF in region i.

Nri: Number of live births in region i.

Equation_6

6.a We will determine the number of LGA/macrosomia attributable to each risk factor for each country by multiplying the adjusted PAF by the total number of LGA/macrosomia in the country.

6.b Regional estimates cases associated with risk factors for all countries in the region i (RFi).


∑number of LGA attributable to each risk factor for each country at regional level 

∑number of macrosomia attributable to each risk factor for each country at regional level 

6.c Global estimates cases associated with RFi across all countdown to 2030 countries.


$$\sum \begin{array}{c} number\;of\;LGA\;attributable\;to\;each\;risk\;factor\;for\;each\;country\;\;level\;\\ at\;all\;Countdown\;to\;2030\;countries\;\; \end{array}$$

$$\sum \begin{array}{c} number\;of\;macrosomia\;attributable\;to\;each\;risk\;factor\;for\;each\;country\;at\;regional\;level\\ at\;all\;Countdown\;to\;2030\;countries\;\; \end{array}$$

Equation_7: Minimum and maximum bounds for PAF.


$$Minimum\;and\;maximum\;PAF\;estimates=PAF_{RFi}\;\times \left(1-Total\;PAF\;from\;other\;RFs\right)$$

RFs: Other Risk factors

### Methodological considerations

For certain risk factors, nationally representative data sources including women of reproductive age will be used in the absence of pregnancy-specific population-level estimates. While these datasets provide standardised and methodologically robust prevalence measures, extrapolation to pregnant populations may introduce proxy bias if exposure distributions differ during pregnancy.

When country-specific prevalence data are unavailable, pooled estimates from high-quality meta-analyses and regional aggregates will be used to enhance parameter stability. Although this approach may reduce contextual specificity and introduce between-study heterogeneity, it is considered methodologically preferable to relying on single-study estimates.

In some included studies, ORs will be converted to RRs to estimate PAFs. Although this conversion is intended to reduce overestimation, it relies on assumptions about baseline outcome prevalence and may still introduce imprecision when outcomes are common, or when the baseline prevalence is mis-specified. Consequently, PAF estimates may be inflated and could potentially overstate the true public health impact compared with estimates derived directly from RRs.

While adjusting gestational diabetes prevalence according to the quality of antenatal care serves as a proxy for facility-level screening and management capacity, it does not capture individual-level factors such as follow-up or treatment adherence that may influence clinical outcomes. Consequently, some estimation bias may persist, as the detection and management of gestational diabetes can vary across settings.

Finally, limiting the review to studies published in English, French and Spanish may introduce language bias by excluding relevant studies published in other languages.

#### Patient and public involvement

This systematic review and meta-analysis protocol aims to analyse the results of existing studies in literature. Patients were therefore not involved in the development of this protocol.

#### Ethical consideration

As this is a systematic review and meta-analysis, ethical approval is not required. We will ensure that all included studies have received ethical approval from relevant institutional or regulatory bodies, as reported by the original authors.

#### Dissemination of results

The results of this systematic review and meta-analysis will be submitted in a peer-reviewed journal. The findings will also be presented in international conferences to engage relevant stakeholders, including policymakers and public health organisations in LMICs, with the aim of informing the development of targeted interventions for reducing the burden of LGA and macrosomia births.

## Supplementary material

10.1136/bmjopen-2025-110407 online supplemental file 1
