# Supplementary material for: Risk factors and population attributable fraction for large-for-gestational-age and macrosomic births in low- and middle-income countries between 2000 and 2025: a protocol for systematic review and meta-analysis
Source: BMJ Open. 2026 May 8;16(5):e110407. doi: 10.1136/bmjopen-2025-110407 (PMC13157752; doi:10.1136/bmjopen-2025-110407)
Supplement: online supplemental file 1 [file bmjopen-16-5-s001.pdf]

SUPPLEMENTARY MATERIALS

Population Concept Context (PCC) approach question:

This study addresses two primary research questions: (1) What are the established risk factors (Concept) associated with large-for-gestational-age (LGA) and macrosomia (Population) in LMICs (Context)? and (2) What proportion of LGA and macrosomia cases can be attributed to key risk factors?

PCC Breakdown:

- Population (P): Infants born LGA (>90th and >97th percentile) or with macrosomia (>4000g and >4500g)
- Concepts (C): Exposure to pre-pregnancy (e.g., pre-pregnancy overweight or obesity, low physical activities, socioeconomic status, parity, birth interval), and pregnancy risk factors (e.g., gestational diabetes, excessive gestational weight gain, access to prenatal care)
- Context (C): Low and middle-income countries (LMICs)

Search strategies

Table S1: PubMed Search Strategy

**PCC approach**	**N°**	**Search equation**	**Results**	**2000-2025**
**Population**	**1**	("High Birth Weight Infant" OR "High Birth Weight Infants" OR "Large for Gestational Age" OR "LGA" OR "birth weight >90th percentile" OR "birth weight >95th percentile" OR "birth weight >97th percentile" OR "Macrosomia" OR "Macrosomias")	11,820	10,366
**Concepts**	**2**	("risk factor" OR "risk factors" OR "associated factor" OR "associated factors" OR "predictor" OR "predictors")	2,053,692	1,827,864
**Context**	**3**	using PubMed LMICs Filter*	2,971,120	2,349,156
**P AND C**	**4**	**1 AND 2**	3,446	3,077
**P AND C AND C**	5	**1 AND 2 AND 3**	892	856

***Pubmed LMICs Filter:**

("emerging country"[tiab] OR "emerging countries"[tiab] OR "emerging nation"[tiab] OR "emerging nations"[tiab] OR "emerging population"[tiab] OR "emerging populations"[tiab] "developing country"[tiab] OR "developing countries"[tiab] OR "developing nation"[tiab] OR "developing nations"[tiab] OR "developing population"[tiab] OR "developing populations"[tiab] OR "developing world"[tiab] OR "less developed country"[tiab] OR "less developed countries"[tiab] OR "less developed nation"[tiab] OR "less developed nations"[tiab] OR "less developed population"[tiab] OR "less developed populations"[tiab] OR "less developed world"[tiab] OR "lesser developed country"[tiab] OR "lesser developed countries"[tiab] OR "lesser developed nation"[tiab] OR "lesser developed nations"[tiab] OR "lesser developed population"[tiab] OR "lesser developed populations"[tiab] OR "lesser developed world"[tiab] OR "under developed country"[tiab] OR "under developed countries"[tiab] OR "under developed nation"[tiab] OR "under developed nations"[tiab] OR "under developed population"[tiab] OR "under developed populations"[tiab] OR "under developed world"[tiab] OR "underdeveloped country"[tiab] OR "underdeveloped countries"[tiab] OR "underdeveloped nation"[tiab] OR "underdeveloped nations"[tiab] OR "underdeveloped population"[tiab] OR "underdeveloped populations"[tiab] OR "underdeveloped world"[tiab] OR "middle income country"[tiab] OR "middle income countries"[tiab] OR "middle income nation"[tiab] OR "middle income nations"[tiab] OR "middle income population"[tiab] OR "middle income populations"[tiab] OR "low income country"[tiab] OR "low income countries"[tiab] OR "low income nation"[tiab] OR "low income nations"[tiab] OR "low income population"[tiab] OR "low income populations"[tiab] OR "lower income country"[tiab] OR "lower income countries"[tiab] OR "lower income nation"[tiab] OR "lower income nations"[tiab] OR "lower income population"[tiab] OR "lower income populations"[tiab] OR "underserved country"[tiab] OR "underserved countries"[tiab] OR "underserved nation"[tiab] OR "underserved nations"[tiab] OR "underserved population"[tiab] OR "underserved populations"[tiab] OR "underserved world"[tiab] OR "under served country"[tiab] OR "under served countries"[tiab] OR "under served nation"[tiab] OR "under served nations"[tiab] OR "under served population"[tiab] OR "under served populations"[tiab] OR "under served world"[tiab] OR "deprived country"[tiab] OR "deprived countries"[tiab] OR "deprived nation"[tiab] OR "deprived nations"[tiab] OR "deprived population"[tiab] OR "deprived populations"[tiab] OR "deprived world"[tiab] OR "poor country"[tiab] OR "poor countries"[tiab] OR "poor nation"[tiab] OR "poor nations"[tiab] OR "poor population"[tiab] OR "poor populations"[tiab] OR "poor world"[tiab] OR "poorer country"[tiab] OR "poorer countries"[tiab] OR "poorer nation"[tiab] OR "poorer nations"[tiab] OR "poorer population"[tiab] OR "poorer populations"[tiab] OR "poorer world"[tiab] OR "developing economy"[tiab] OR "developing economies"[tiab] OR "less developed economy"[tiab] OR "less developed economies"[tiab] OR "lesser developed economy"[tiab] OR "lesser developed economies"[tiab] OR "under developed economy"[tiab] OR "under developed economies"[tiab] OR "underdeveloped economy"[tiab] OR "underdeveloped economies"[tiab] OR "middle income economy"[tiab] OR "middle income economies"[tiab] OR "low income economy"[tiab] OR "low income economies"[tiab] OR "lower income economy"[tiab] OR "lower income economies"[tiab] OR "low gdp"[tiab] OR "low gnp"[tiab] OR "low gross domestic"[tiab] OR "low gross national"[tiab] OR "lower gdp"[tiab] OR "lower gnp"[tiab] OR "lower gross domestic"[tiab] OR "lower gross national"[tiab] OR lmic[tiab] OR lmics[tiab] OR "third world"[tiab] OR "lami country"[tiab] OR "lami countries"[tiab] OR "transitional country"[tiab] OR "transitional countries"[tiab] OR Africa[tiab] OR Asia[tiab] OR Caribbean[tiab] OR "West Indies"[tiab] OR "South America"[tiab] OR "Latin America"[tiab] OR "Central America"[tiab] OR "Atlantic Islands"[tiab] OR "Commonwealth of Independent States"[tiab] OR "Pacific Islands"[tiab] OR "Indian Ocean Islands"[tiab] OR "Eastern Europe"[tiab] OR Afghanistan[tiab] OR Albania[tiab] OR Algeria[tiab] OR Angola[tiab] OR Armenia[tiab] OR Armenian[tiab] OR Azerbaijan[tiab] OR Bangladesh[tiab] OR Benin[tiab] OR Byelarus[tiab] OR Byelorussian[tiab] OR Belarus[tiab] OR Belorussian[tiab] OR Belorussia[tiab] OR Belize[tiab] OR Bhutan[tiab] OR Bolivia[tiab] OR Bosnia[tiab] OR Herzegovina[tiab] OR Hercegovina[tiab] OR Botswana[tiab] OR Brasil[tiab] OR Brazil[tiab] OR Bulgaria[tiab] OR "Burkina Faso"[tiab] OR "Burkina Fasso"[tiab] OR "Upper Volta"[tiab] OR Burundi[tiab] OR Urundi[tiab] OR Cambodia[tiab] OR "Khmer

Republic"[tiab] OR Kampuchea[tiab] OR Cameroon[tiab] OR Cameroons[tiab] OR Cameron[tiab] OR "Cape Verde"[tiab] OR "Central African Republic"[tiab] OR Chad[tiab] OR China[tiab] OR Colombia[tiab] OR Comoros[tiab] OR "Comoro Islands"[tiab] OR Comores[tiab] OR Mayotte[tiab] OR Congo[tiab] OR Zaire[tiab] OR "Costa Rica"[tiab] OR "Cote d'Ivoire"[tiab] OR "Ivory Coast"[tiab] OR Cuba[tiab] OR Czechoslovakia[tiab] OR Slovakia[tiab] OR Djibouti[tiab] OR "French Somaliland"[tiab] OR Dominica[tiab] OR Dominican Republic[tiab] OR "East Timor"[tiab] OR "East Timur"[tiab] OR "Timor Leste"[tiab] OR Ecuador[tiab] OR Egypt[tiab] OR "El Salvador"[tiab] OR Eritrea[tiab] OR Ethiopia[tiab] OR Fiji[tiab] OR Gabon[tiab] OR "Gabonese Republic"[tiab] OR Gambia[tiab] OR Gaza[tiab] OR "Georgia Republic"[tiab] OR "Georgian Republic"[tiab] OR Ghana[tiab] OR "Gold Coast"[tiab] OR Grenada[tiab] OR Guatemala[tiab] OR Guinea[tiab] OR Guiana[tiab] OR Guyana[tiab] OR Haiti[tiab] OR Honduras[tiab] OR India[tiab] OR Maldives[tiab] OR Indonesia[tiab] OR Iran[tiab] OR Iraq[tiab] OR Jamaica[tiab] OR Jordan[tiab] OR Kazakhstan[tiab] OR Kazakh[tiab] OR Kenya[tiab] OR Kiribati[tiab] OR Korea[tiab] OR Kosovo[tiab] OR Kyrgyzstan[tiab] OR Kirghizia[tiab] OR Kyrgyz Republic[tiab] OR Kirghiz[tiab] OR Kirgizstan[tiab] OR "Lao PDR"[tiab] OR Laos[tiab] OR Lebanon[tiab] OR Lesotho[tiab] OR Basutoland[tiab] OR Liberia[tiab] OR Libya[tiab] OR Macedonia[tiab] OR Madagascar[tiab] OR "Malagasy Republic"[tiab] OR Malaysia[tiab] OR Malaya[tiab] OR Malay[tiab] OR Sabah[tiab] OR Sarawak[tiab] OR Malawi[tiab] OR Nyasaland[tiab] OR Mali[tiab] OR "Marshall Islands"[tiab] OR Mauritania[tiab] OR Mauritius[tiab] OR "Agalega Islands"[tiab] OR "Melanesia"[tiab] OR Mexico[tiab] OR Micronesia[tiab] OR "Middle East"[tiab] OR Moldova[tiab] OR Moldovia[tiab] OR Moldovian[tiab] OR Mongolia[tiab] OR Montenegro[tiab] OR Morocco[tiab] OR Ifni[tiab] OR Mozambique[tiab] OR Myanmar[tiab] OR Myanma[tiab] OR Burma[tiab] OR Namibia[tiab] OR Nepal[tiab] OR Nicaragua[tiab] OR Niger[tiab] OR Nigeria[tiab] OR Muscat[tiab] OR Pakistan[tiab] OR Palau[tiab] OR Palestine[tiab] OR Panama[tiab] OR Paraguay[tiab] OR Peru[tiab] OR Philippines[tiab] OR Philipines[tiab] OR Phillipines[tiab] OR Phillippines[tiab] OR Romania[tiab] OR Rumania[tiab] OR Roumania[tiab] OR Rwanda[tiab] OR Ruanda[tiab] OR Saint Kitts[tiab] OR "St Kitts"[tiab] OR Nevis[tiab] OR "Saint Lucia"[tiab] OR "St Lucia"[tiab] OR "Saint Vincent"[tiab] OR "St Vincent"[tiab] OR Grenadines[tiab] OR Samoa[tiab] OR "Samoan Islands"[tiab] OR "Navigator Island"[tiab] OR "Navigator Islands"[tiab] OR "Sao Tome"[tiab] OR Senegal[tiab] OR Serbia[tiab] OR Montenegro[tiab] OR "Sierra Leone"[tiab] OR Sri Lanka[tiab] OR Ceylon[tiab] OR "Solomon Islands"[tiab] OR Somalia[tiab] OR Sudan[tiab] OR Suriname[tiab] OR Surinam[tiab] OR Swaziland[tiab] OR Syria[tiab] OR Syrian[tiab] OR Tajikistan[tiab] OR Tadzhikistan[tiab] OR Tadjikistan[tiab] OR Tadzhik[tiab] OR Tanzania[tiab] OR Thailand[tiab] OR Togo[tiab] OR "Togolese Republic"[tiab] OR Tonga[tiab] OR Tunisia[tiab] OR Turkey[tiab] OR Turkmenistan[tiab] OR Turkmen[tiab] OR Tuvalu[tiab] OR Uganda[tiab] OR Ukraine[tiab] OR Uzbekistan[tiab] OR Uzbek[tiab] OR Vanuatu[tiab] OR "New Hebrides"[tiab] OR Vietnam[tiab] OR "Viet Nam"[tiab] OR "West Bank"[tiab] OR Yemen[tiab] OR Yugoslavia[tiab] OR Zambia[tiab] OR Zimbabwe[tiab] OR Rhodesia[tiab] OR "Developing Countries"[Mesh] OR Africa[Mesh] OR Asia[Mesh] OR "South America"[Mesh] OR "Latin America"[Mesh] OR "Central America"[Mesh] OR "Atlantic Islands"[Mesh] OR "Commonwealth of Independent States"[Mesh:NoExp] OR "Pacific Islands"[Mesh] OR "Indian Ocean Islands"[Mesh] OR "Europe, Eastern"[Mesh] OR "Southern African Development Community"[tiab] OR "East African Community"[tiab] OR "West African Health Organisation"[tiab] OR "Sub Saharan Africa"[tiab] OR "SubSaharan Africa"[tiab])

Table S2: ProQuest Central and Scopus search Strategy

PCC approach	N°	Search equation	Scopus (Ti-Ab-Key)		ProQuest Central (NOFT)	
			Results	2000-2025	Results	2000-2025
**Population**	**1**	("High Birth Weight Infant" OR "High Birth Weight Infants" OR "Large for Gestational Age" OR "LGA" OR "birth weight >90th percentile" OR "birth weight >95th percentile" OR "birth weight >97th percentile" OR "Macrosomia" OR "Macrosomias")	18683	16653	19350	18984
**Concepts**	**2**	("risk factor" OR "risk factors" OR "associated factor" OR "associated factors" OR "predictor" OR "predictors")	2,854,563	2,584,769	1 357 927	1 303 664
**Context**	**3**	using LMICs Filter for ProQuest Central and Scopus database**	7,588,291	6,553,704	149 219 342	141 199 062
**P AND C**	**4**	**1 AND 2**	5,624	5,215	1 331	1 304
**P AND C AND C**	5	**1 AND 2 AND 3**	1,167	1,133	469	468

**LMICs filter for ProQuest Central and Scopus database

("emerging country" OR "emerging countries" OR "emerging nation" OR "emerging nations" OR "emerging population" OR "emerging populations" OR "developing country" OR "developing countries" OR "developing nation" OR "developing nations" OR "developing population" OR "developing populations" OR "developing world" OR "less developed country" OR "less developed countries" OR "less developed nation" OR "less developed nations" OR "less developed population" OR "less developed populations" OR "less developed world" OR "lesser developed country" OR "lesser developed countries" OR "lesser developed nation" OR "lesser developed nations" OR "lesser developed population" OR "lesser developed populations" OR "lesser developed world" OR "under developed country" OR "under developed countries" OR "under developed nation" OR "under developed nations" OR "under developed population" OR "under developed populations" OR "under developed world" OR "underdeveloped country" OR "underdeveloped countries" OR "underdeveloped nation" OR "underdeveloped nations" OR "underdeveloped population" OR "underdeveloped populations" OR "underdeveloped world" OR "middle income country" OR "middle income countries" OR "middle income nation" OR "middle income nations" OR "middle income population" OR "middle income populations" OR "low income country" OR "low income countries" OR "low income nation" OR "low income nations" OR "low income population" OR "low income populations" OR "lower income country" OR "lower income countries" OR "lower income nation" OR "lower income nations" OR "lower income population" OR "lower income populations" OR "underserved country" OR "underserved countries" OR "underserved nation" OR "underserved nations" OR "underserved population" OR "underserved populations" OR "underserved world" OR "under served country" OR "under served countries" OR "under served nation" OR "under served nations" OR "under served population" OR "under served populations" OR "under served world" OR "deprived country" OR "deprived countries" OR "deprived nation" OR "deprived nations" OR "deprived population" OR "deprived populations" OR "deprived world" OR "poor country" OR "poor countries" OR "poor nation" OR "poor nations" OR "poor population" OR "poor populations" OR "poor world" OR "poorer country" OR "poorer countries" OR "poorer nation" OR "poorer nations" OR "poorer population" OR "poorer populations" OR "poorer world" OR "developing economy" OR "developing economies" OR "less developed economy" OR "less developed economies" OR "lesser developed economy" OR "lesser developed economies" OR "under developed economy" OR "under developed economies" OR "underdeveloped economy" OR "underdeveloped economies" OR "middle income economy" OR "middle income economies" OR "low income economy" OR "low income economies" OR "lower income economy" OR "lower income economies" OR "low gdp" OR "low gnp" OR "low gross domestic" OR "low gross national" OR "lower gdp" OR "lower gnp" OR "lower gross domestic" OR "lower gross national" OR "lmic" OR "lmics" OR "third world" OR "lami country" OR "lami countries" OR "transitional country" OR "transitional countries" OR "Africa" OR "Asia" OR "Caribbean" OR "West Indies" OR "South America" OR "Latin America" OR "Central America" OR "Atlantic Islands" OR "Commonwealth of Independent States" OR "Pacific Islands" OR "Indian Ocean Islands" OR "Eastern Europe" OR "Afghanistan" OR "Albania" OR "Algeria" OR "Angola" OR "Armenia" OR "Armenian" OR "Azerbaijan" OR "Bangladesh" OR "Benin" OR "Byelarus" OR "Byelorussian" OR "Belarus" OR "Belorussian" OR "Belorussia" OR "Belize" OR "Bhutan" OR "Bolivia" OR "Bosnia" OR "Herzegovina" OR "Hercegovina" OR "Botswana" OR "Brasil" OR "Brazil" OR "Bulgaria" OR "Burkina Faso" OR "Burkina Fasso" OR "Upper Volta" OR "Burundi" OR "Urundi" OR "Cambodia" OR "Khmer Republic" OR "Kampuchea" OR "Cameroon" OR "Cameroons" OR "Cameron" OR "Cape Verde" OR "Central African Republic" OR "Chad" OR "China" OR "Colombia" OR "Comoros" OR "Comoro Islands" OR "Comores" OR "Mayotte" OR "Congo" OR "Zaire" OR "Costa Rica" OR "Cote d'Ivoire" OR "Ivory Coast" OR "Cuba" OR "Czechoslovakia" OR "Slovakia" OR "Djibouti" OR "French Somaliland" OR "Dominica" OR "Dominican Republic" OR "East Timor" OR "East Timur" OR "Timor Leste" OR "Ecuador" OR "Egypt" OR "El Salvador" OR "Eritrea" OR "Ethiopia" OR "Fiji" OR "Gabon" OR "Gabonese Republic" OR "Gambia" OR "Gaza" OR "Georgia Republic" OR "Georgian Republic" OR "Ghana" OR "Gold Coast" OR "Grenada" OR "Guatemala" OR "Guinea" OR "Guiana" OR "Guyana" OR "Haiti" OR "Honduras" OR "India" OR "Maldives" OR "Indonesia" OR "Iran" OR "Iraq" OR "Jamaica"

OR "Jordan" OR "Kazakhstan" OR "Kazakh" OR "Kenya" OR "Kiribati" OR "Korea" OR "Kosovo" OR "Kyrgyzstan" OR "Kirghizia" OR "Kyrgyz Republic" OR "Kirghiz" OR "Kirgizstan" OR "Lao PDR" OR "Laos" OR "Lebanon" OR "Lesotho" OR "Basutoland" OR "Liberia" OR "Libya" OR "Macedonia" OR "Madagascar" OR "Malagasy Republic" OR "Malaysia" OR "Malaya" OR "Malay" OR "Sabah" OR "Sarawak" OR "Malawi" OR "Nyasaland" OR "Mali" OR "Marshall Islands" OR "Mauritania" OR "Mauritius" OR "Agalega Islands" OR "Melanesia" OR "Mexico" OR "Micronesia" OR "Middle East" OR "Moldova" OR "Moldovia" OR "Moldovian" OR "Mongolia" OR "Montenegro" OR "Morocco" OR "Ifni" OR "Mozambique" OR "Myanmar" OR "Myanma" OR "Burma" OR "Namibia" OR "Nepal" OR "Nicaragua" OR "Niger" OR "Nigeria" OR "Muscat" OR "Pakistan" OR "Palau" OR "Palestine" OR "Panama" OR "Paraguay" OR "Peru" OR "Philippines" OR "Philipines" OR "Phillipines" OR "Phillippines" OR "Romania" OR "Rumania" OR "Roumania" OR "Rwanda" OR "Ruanda" OR "Saint Kitts" OR "St Kitts" OR "Nevis" OR "Saint Lucia" OR "St Lucia" OR "Saint Vincent" OR "St Vincent" OR "Grenadines" OR "Samoa" OR "Samoan Islands" OR "Navigator Island" OR "Navigator Islands" OR "Sao Tome" OR "Senegal" OR "Serbia" OR "Montenegro" OR "Sierra Leone" OR "Sri Lanka" OR "Ceylon" OR "Solomon Islands" OR "Somalia" OR "Sudan" OR "Suriname" OR "Surinam" OR "Swaziland" OR "Syria" OR "Syrian" OR "Tajikistan" OR "Tadzhikistan" OR "Tadjikistan" OR "Tadzhik" OR "Tanzania" OR "Thailand" OR "Togo" OR "Togolese Republic" OR "Tonga" OR "Tunisia" OR "Turkey" OR "Turkmenistan" OR "Turkmen" OR "Tuvalu" OR "Uganda" OR "Ukraine" OR "Uzbekistan" OR "Uzbek" OR "Vanuatu" OR "New Hebrides" OR "Vietnam" OR "Viet Nam" OR "West Bank" OR "Yemen" OR "Yugoslavia" OR "Zambia" OR "Zimbabwe" OR "Rhodesia" OR "Developing Countries" OR "Asia" OR "Caribbean Region" OR "West Indies" OR "South America" OR "Latin America" OR "Central America" OR "Atlantic Islands" OR "Commonwealth of Independent States" OR "Pacific Islands" OR "Indian Ocean Islands" OR "Southern African Development Community" OR "East African Community" OR "West African Health Organisation")

Table S3: Search strategy in local databases

Approach PCC	Search equation	Africa index medicus (Title, abstract, subject)	Index medicus for the south easth Asia (Title, abstract, subject)	Latine america and carribbean literature of health sciences (Title, abstract, subject)
Population (P)	("High Birth Weight Infant" OR "High Birth Weight Infants" OR "Large for Gestational Age" OR "LGA" OR "birth weight >90th percentile" OR "birth weight >95th percentile" OR "birth weight >97th percentile" OR "Macrosomia" OR "Macrosomias")	66	152	494
Concepts (C)	("risk factor" OR "risk factors" OR "associated factor" OR "associated factors" OR "predictor" OR "predictors")	1622	12570	59343
P AND C	("High Birth Weight Infant" OR "High Birth Weight Infants" OR "Large for Gestational Age" OR "LGA" OR "birth weight >90th percentile" OR "birth weight >95th percentile" OR "birth weight >97th percentile" OR "Macrosomia" OR "Macrosomias") AND ("risk factor" OR "risk factors" OR "associated factor" OR "associated factors" OR "predictor" OR "predictors")	11	36	152

Table S4. Estimation of prevalence of potential key risk factors of LGA/macrosomia to be included in this study

Exposure period	Risk factors	Prevalence estimate method	Source	Publication year	Type of studies
Pre-pregnancy factors	Parity (≥3)	Country specific	DHS	Most recent	National representative survey
	Birth interval (< 2 years)	Country specific	DHS	Most recent	National representative survey
	Mother's age at birth (≥ 35 years)	Country specific	DHS	Most recent	National representative survey
	Body mass index (BMI≥ 25 or ≥30)	Country specific	DHS or IHME	Most recent	National representative survey
	Low physical activity	Country specific	IHME	Most recent	National representative survey
	Residence (rural vs urban)	Country specific	DHS	Most recent	National representative survey
	Income	Country specific	DHS	Most recent	National representative survey
Pregnancy factors	Antenatal care follow-up (< 4 or < 8)	Country specific	DHS	Most recent	National representative survey
	Male fetus	Country specific	DHS	Most recent	National representative survey
	Gestational diabetes	Regional	Ye et al. [1]	2022	Systematic review and meta-analysis

Excessive weight gain over pregnancy	Regional	Goldstein et al. [2]	2017	Systematic review and meta-analysis
Pre-existing diabetes in pregnancy	Regional	Chivese et al. [3]	2021	Systematic review and meta-analysis
Overnutrition or hypercaloric diet	Regional			estimates generated by IHME
History of delivering a macrosomic baby	Regional			Systematic review and meta-analysis
post-term gestation (>41 weeks)	Regional			Systematic review and meta-analysis
Missed screening for gestational diabetes	Regional			Systematic review and meta-analysis
Lack of nutritional counseling	Regional			Systematic review and meta-analysis

Table S5: PRISMA-P (Preferred Reporting Items for Systematic review and Meta-Analysis Protocols) 2015 checklist: recommended items to address in a systematic review protocol[4]

Section and topic	Item No	Checklist item	Location where item is reported
**ADMINISTRATIVE INFORMATION**			
Title:			
Identification	1a	Identify the report as a protocol of a systematic review	Page 1
Update	1b	If the protocol is for an update of a previous systematic review, identify as such	NA
Registration	2	If registered, provide the name of the registry (such as PROSPERO) and registration number	Page 2
Authors:			
Contact	3a	Provide name, institutional affiliation, e-mail address of all protocol authors; provide physical mailing address of corresponding author	Page 1
Contributions	3b	Describe contributions of protocol authors and identify the guarantor of the review	Page 17
Amendments	4	If the protocol represents an amendment of a previously completed or published protocol, identify as such and list changes; otherwise, state plan for documenting important protocol amendments	NA
Support:			
Sources	5a	Indicate sources of financial or other support for the review	NA
Sponsor	5b	Provide name for the review funder and/or sponsor	NA
Role of sponsor or funder	5c	Describe roles of funder(s), sponsor(s), and/or institution(s), if any, in developing the protocol	NA
**INTRODUCTION**			
Rationale	6	Describe the rationale for the review in the context of what is already known	Page 5-6
Objectives	7	Provide an explicit statement of the question(s) the review will address with reference to participants, interventions, comparators, and outcomes (PICO)	Page 6-7
**METHODS**			
Eligibility criteria	8	Specify the study characteristics (such as PICO, study design, setting, time frame) and report characteristics (such as years considered, language, publication status) to be used as criteria for eligibility for the review	Page 6-7
Information sources	9	Describe all intended information sources (such as electronic databases, contact with study authors, trial registers or other grey literature sources) with planned dates of coverage	Page 6-7
Search strategy	10	Present draft of search strategy to be used for at least one electronic database, including planned limits, such that it could be repeated	Page 6-7
Study records:			
Data management	11a	Describe the mechanism(s) that will be used to manage records and data throughout the review	Page 8
Selection process	11b	State the process that will be used for selecting studies (such as two independent reviewers) through each phase of the review (that is, screening, eligibility and inclusion in meta-analysis)	Page 8
Data collection process	11c	Describe planned method of extracting data from reports (such as piloting forms, done independently, in duplicate), any processes for obtaining and confirming data from investigators	Page 7
Data items	12	List and define all variables for which data will be sought (such as PICO items, funding sources), any pre-planned data assumptions and simplifications	Page 9-10
Outcomes and prioritization	13	List and define all outcomes for which data will be sought, including prioritization of main and additional outcomes, with rationale	Page 10-11

Risk of bias in individual studies	14	Describe anticipated methods for assessing risk of bias of individual studies, including whether this will be done at the outcome or study level, or both; state how this information will be used in data synthesis	Page 9
Data synthesis	15a	Describe criteria under which study data will be quantitatively synthesised	Page 11-13
	15b	If data are appropriate for quantitative synthesis, describe planned summary measures, methods of handling data and methods of combining data from studies, including any planned exploration of consistency (such as I^2, Kendall's τ)	Page 11-13
	15c	Describe any proposed additional analyses (such as sensitivity or subgroup analyses, meta-regression)	Page 12
	15d	If quantitative synthesis is not appropriate, describe the type of summary planned	NA
Meta-bias(es)	16	Specify any planned assessment of meta-bias(es) (such as publication bias across studies, selective reporting within studies)	Page 10-11
Confidence in cumulative evidence	17	Describe how the strength of the body of evidence will be assessed (such as GRADE)	Page 5, 13-15

References for supplementary materials

1. Ye W, Luo C, Huang J, Li C, Liu Z, Liu F. Gestational diabetes mellitus and adverse pregnancy outcomes: systematic review and meta-analysis. BMJ. 2022 May 25;377:e067946. doi:10.1136/bmj-2021-067946 PubMed PMID: 35613728; PubMed Central PMCID: PMC9131781.

2. Goldstein RF, Abell SK, Ranasinha S, Misso M, Boyle JA, Black MH, et al. Association of Gestational Weight Gain With Maternal and Infant Outcomes: A Systematic Review and Meta-analysis. JAMA. 2017 Jun 6;317(21):2207–25. doi:10.1001/jama.2017.3635

3. Chivese T, Hoegfeldt CA, Werfalli M, Yuen L, Sun H, Karuranga S, et al. IDF Diabetes Atlas: The prevalence of pre-existing diabetes in pregnancy – A systematic review and meta-analysis of studies published during 2010–2020. Diabetes Research and Clinical Practice. 2022 Jan 1;183. doi:10.1016/j.diabres.2021.109049 PubMed PMID: 34883190.

4. Shamseer L, Moher D, Clarke M, Ghersi D, Liberati A, Petticrew M, et al. Preferred reporting items for systematic review and meta-analysis protocols (PRISMA-P) 2015: elaboration and explanation. BMJ. 2015 Jan 2;350:g7647. doi:10.1136/bmj.g7647 PubMed PMID: 25555855.
