# Supplementary material for: Risk factors and population attributable fraction for large-for-gestational-age and macrosomic births in low- and middle-income countries between 2000 and 2025: a protocol for systematic review and meta-analysis
Source: BMJ Open. 2026 May 8;16(5):e110407. doi: 10.1136/bmjopen-2025-110407 (PMC13157752; doi:10.1136/bmjopen-2025-110407)
Supplement: Supplementary file 2 [file bmjopen-2025-110407.reviewer_comments.pdf]

PEER REVIEW HISTORY

BMJ Open publishes all reviews undertaken for accepted manuscripts. Reviewers are asked to complete a checklist review form and are provided with free text boxes to elaborate on their assessment. These free text comments are reproduced below.

ARTICLE DETAILS

Title (Provisional)

Risk factors and population attributable fraction for large-for-gestational-age and macrosomic births in low-and middle-income countries between 2000 and 2025: A Protocol for Systematic Review and Meta-Analysis

Authors

Kirakoya-Samadoulougou, Fati; Blencowe, Hannah; Ilboudo, Dieudonné; Ukwishaka, Joyeuse; Suarez Idueta, Lorena; Hazel , Elizabeth A; Ohuma, Eric; Erchick, Daniel Joseph; Katz, Joanne; Lee, Anne C; Black, Robert E.

VERSION 1 - REVIEW

Reviewer 1

Name Facchinetti , Fabio

Affiliation Azienda Ospedaliera Universitaria Policlinico di Modena, Obstetric Unit, Mother Infant Department

Date 28-Jan-2026

COI None

The planned review and Metaanalysis on risk factors for big babies in LMIC is of interest. Iin addition it is very important to evaluate the population attributed risk factors since this info could guide specific interventions.

The study is supported by adequate and recent biblography and demonstrated to be able to catch -up approriiate info from the literature, in order to reach the goal.

Methodologically the study reach standard requirements.

The only remrks is about ponsorship. Even if the Authros declare not having "external support" tehy should any way acknowledge their Institutions.

Reviewer 2

Name Mengesha, Meresa Berwo

Affiliation Adigrat University College of Health Sciences, Midwifery

Date 15-Feb-2026

COI **None**

General comments

1. Generally, determining the country-specific PAF for each risk factor is really advisable and is one of the strongest sides of the protocol.

2. Strengths and limitations of this study, section; The study will use a systematic search strategy, including multiple databases to ensure thorough coverage of the topic. However, Limited databases: Only three (Medline, Scopus, ProQuest Central); Embase, Web of Science, or regional (e.g., LILACS for Latin America) could capture more LMIC studies. Narrow database scope; no grey literature beyond references.

3. Prevalence sources rely on non-pregnant women data (DHS/IHME) for some factors, regional averages for gaps, and external meta-analyses (e.g., Ye 2022 for GDM), introducing proxy bias; OR-to-RR conversion assumes known outcome prevalence in unexposed, which may inflate PAFs for common outcomes like LGA (~10%).

4. Inclusion (observational, RCTs, reviews) without specifying gestational age standards (e.g., INTERGROWTH-21st vs. local); subgroup plans (90th/97th percentile, ultrasound/LMP) are good but may leave residual confounding.

5. OR approximation bias; no GRADE for evidence certainty

Specific comments

1. The authors acknowledge that using odds ratios (ORs) instead of relative risks (RRs) for common outcomes can inflate PAF estimates. While they provide a conversion formula, its accuracy depends on knowing the "prevalence of LGA and macrosomia in the reference group which may not be consistently reported across all primary studies. The authors need to clarify and justify and be cautious about this issue.

2. The protocol notes that PAF estimates may be more relevant for specific geographic regions and may not extend fully to all LMICs. Using regional averages for countries lacking national survey data could mask significant local variations in risk factor prevalence. The authors need to consider and acknowledge this before the actual data collection.

3. The search strategy covers literature from the year 2000 to 2025. Given rapid changes in nutrition and health systems in many LMICs, data from 20-25 years ago may no longer accurately reflect current risk landscapes. How the authors clarify this?

4. Although the protocol includes multiple standard definitions for LGA (90th vs. 97th percentile) and macrosomia (≥4000g vs. ≥4500g), the wide variety of thresholds used in primary studies can lead to significant clinical and statistical heterogeneity in meta-analyses.

5. The decision to exclude meta-analysis for risk factors reported in fewer than three studies is statistically sound but may result in the omission of emerging or contextually important modifiable factors that are currently under-researched.

6. The formula for adjusting the prevalence of gestational diabetes based on "ANC quality" (defined as the percentage of facilities willing to screen) is a creative approach, but it may oversimplify the actual clinical management and treatment adherence of pregnant women in practice.

7. By only including studies that provide quantitative data on risk factors, the review may miss qualitative insights into the drivers of these risk factors (e.g., cultural beliefs regarding "eating for two" or barriers to physical activity). The review may be benefited if it plans also to include these barriers in the analysis plan.

8. The estimation of PAFs is limited only to countries included in the "Countdown to 2030" initiative. This excludes other LMICs that may also face a high burden of LGA or macrosomia but fall outside this specific global health group.

VERSION 1 - AUTHOR RESPONSE

Reviewer:1
Dr. Fabio Facchinetti , Azienda Ospedaliera Universitaria Policlinico di Modena
Comments to the Author:

The planned review and Meta-analysis on risk factors for big babies in LMIC is of interest. In addition, it is very important to evaluate the population attributed risk factors since this info could guide specific interventions.

The study is supported by adequate and recent biblography and demonstrated to be able to catch -up appropriate info from the literature, in order to reach the goal. Methodologically, the study reaches standard requirements.

Comment 1: The only remark is about sponsorship. Even if the Authors declare not having "external support" they should any way acknowledge their Institutions.
Reponses: Thank you for this comment. We have mentioned the institutional affiliations of the various authors and have gratefully acknowledged our institutions in the Acknowledgments section. As follow (on line 379-380): *“The authors gratefully acknowledge their institutions for providing the necessary resources and a supportive environment to conduct this study.”*

Reviewer: 2
Mr. Meresa Berwo Mengesha, Adigrat University College of Health Sciences
Comments to the Author:

General comments

Generally, determining the country-specific PAF for each risk factor is really advisable and is one of the strongest sides of the protocol.

Comment 1. Strengths and limitations of this study, section; The study will use a systematic search strategy, including multiple databases to ensure thorough coverage of the topic.

However, Limited databases: Only three (Medline, Scopus, ProQuest Central); Embase, Web of Science, or regional (e.g., LILACS for Latin America) could capture more LMIC studies. Narrow database scope; no grey literature beyond references.

Reponses: Thank you for this comment. The selection of Medline, Scopus, and ProQuest Central was carefully considered to ensure broad and multidisciplinary coverage of peer-reviewed biomedical and public health literature. These databases were chosen because of their substantial overlap in indexing international research, including studies conducted in low- and middle-income countries (LMICs), while also providing complementary coverage across clinical, epidemiological, and social science domains.

In addition to these three databases, we will also consider the local databases provided (Africa index Medicus, Index Medicus for the south Asia and Latine America and Caribbean literature of health sciences) (on line 129-131): "*The search will be conducted across three major databases (Medline, Scopus, and ProQuest Central). We will also explore local databases such as: Africa index Medicus, Index Medicus for the south Asia, and Latine America and Caribbean literature of health sciences.*"

Comment 3. Prevalence sources rely on non-pregnant women data (DHS/IHME) for some factors, regional averages for gaps, and external meta-analyses (e.g., Ye 2022 for GDM), introducing proxy bias; OR-to-RR conversion assumes known outcome prevalence in unexposed, which may inflate PAFs for common outcomes like LGA (~10%).

Reponses: Thank you for these comments. Regarding the use of prevalence estimates derived from women of reproductive age rather than exclusively pregnant women, we have clarified this assumption in the Limitations section. The following sentence has been added (on line 330-333): "*For certain risk factors, nationally representative data sources including women of reproductive age will be used in the absence of pregnancy-specific population-level estimates. While these datasets provide standardized and methodologically robust prevalence measures, extrapolation to pregnant populations may introduce proxy bias if exposure distributions differ during pregnancy*."

Concerning the use of regional aggregates and external meta-analyses for specific exposures such as gestational diabetes mellitus, we have added the following clarification (on line 334-337): "*When country-specific prevalence data are unavailable, pooled estimates from high-quality meta-analyses and regional aggregates will be used to enhance parameter stability. Although this approach may reduce contextual specificity and introduce between-study heterogeneity, it was considered methodologically preferable to relying on single-study estimates*."

Regarding the risk of PAF inflation and the conversion of odds ratios (ORs) to relative risks (RRs), we have strengthened the methodological description and explicitly acknowledged the associated assumptions. The manuscript now states (on line 338-345): "*In some included studies, odds ratios (ORs) will be converted to relative risks (RRs) to estimate PAFs. Although this conversion is intended to reduce overestimation, it relies on assumptions about baseline outcome prevalence and may still introduce imprecision when outcomes are common or when baseline prevalence is mis-specified. Consequently, PAF estimates may be inflated and could*

potentially overstate the true public health impact compared with estimates derived directly from relative risks."

These additions have been incorporated to improve transparency regarding modeling assumptions and to explicitly acknowledge potential sources of bias.

Comment 4. Inclusion (observational, RCTs, reviews) without specifying gestational age standards (e.g., INTERGROWTH-21st vs. local); subgroup plans (90th/97th percentile, ultrasound/LMP) are good but may leave residual confounding.

Reponses: Thank you for these comments. We agree that variability in gestational age assessment standards and fetal growth references may introduce heterogeneity and that despite conducting subgroup analyses potential residual confounding may remain due to within-subgroup variability. To address this, we have clarified the analysis strategy in the revised manuscript (on line 291-298): "*In subgroup analyses, we will compare common thresholds for LGA (90th vs. 97th percentile) and macrosomia (≥4000 g vs. ≥4500 g). We will also compare studies using INTERGROWTH versus other growth references and conduct stratified analyses by gestational age assessment method (ultrasound vs. LMP), where reported. While these stratifications help account for methodological differences, residual confounding may persist due to within-subgroup variability in gestational age assessment, growth references, and outcome definitions, as well as heterogeneity in measurement accuracy across settings. This variability may slightly affect the comparability of effect estimates and attributable fractions.*

Comment 5. OR approximation bias; no GRADE for evidence certainly

Reponses: Thank you for this comment. We have clarified the potential OR approximation bias in the manuscript as noted in the limitations section, ORs will be converted to RRs for PAF estimation to reduce overestimation, though residual imprecision may remain for common outcomes (on line 338-343): "*In some included studies, odds ratios (ORs) will be converted to relative risks (RRs) to estimate PAFs. Although this conversion is intended to reduce overestimation, it relies on assumptions about baseline outcome prevalence and may still introduce imprecision when outcomes are common, or when the baseline prevalence is mis-specified consequently, PAF estimates may be inflated and could potentially overstate the true public health impact compared with estimates derived directly from relative risks.*"

A formal GRADE assessment was not conducted due to the modeling design and reliance on published pooled estimates; this may limit formal evaluation of evidence certainty.

Specific comments

Comment 1. The authors acknowledge that using odds ratios (ORs) instead of relative risks (RRs) for common outcomes can inflate PAF estimates. While they provide a conversion formula, its accuracy depends on knowing the "prevalence of LGA and macrosomia in the reference group which may not be consistently reported across all primary studies. The authors need to clarify and justify and be cautious about this issue.

Reponses: Thank you for this comment. We will use odds ratios (ORs) from primary studies and convert them to relative risks (RRs) to estimate Population Attributable Fractions (PAFs). While we acknowledge that the accuracy of this conversion depends on baseline prevalence in the reference (unexposed) group which may not consistently be reported across all studies, this method provides a practical and standardized way to harmonize effect measures across heterogeneous data sources In our protocol, we proposed this (on line 266-270): "*After identifying relative risks (RRs) or odds ratios (ORs) for each risk factor of LGA/macrosomia from the included studies, ORs will be converted to RRs using the prevalence of LGA/macrosomia in the reference group. If this prevalence is not reported, we will use the most recent national prevalence; if unavailable, the overall regional prevalence (Africa, Asia, or Latin America) will be used*."

We also report the limitations of this approach in the methodology section (on line 338-343): "*In some included studies, odds ratios (ORs) will be converted to relative risks (RRs) to estimate PAFs. Although this conversion is intended to reduce overestimation, it relies on assumptions about baseline outcome prevalence and may still introduce imprecision when outcomes are common, or when the baseline prevalence is mis-specified. Consequently, PAF estimates may be inflated and could potentially overstate the true public health impact compared with estimates derived directly from relative risks*."

Comment 2. The protocol notes that PAF estimates may be more relevant for specific geographic regions and may not extend fully to all LMICs. Using regional averages for countries lacking national survey data could mask significant local variations in risk factor prevalence. The authors need to consider and acknowledge this before the actual data collection.

Reponses: Thank you for this comment. We acknowledge that using regional averages may obscure local heterogeneity in risk factor prevalence. This limitation has been explicitly acknowledged in the Limitations section, and caution is advised when interpreting PAFs for countries without primary national data (on line 334-337): "*When country-specific prevalence data are unavailable, pooled estimates from high-quality meta-analyses and regional aggregates will be used to enhance parameter stability. Although this approach may reduce contextual specificity and introduce between-study heterogeneity, it is considered methodologically preferable to relying on single-study estimates*."

Comment 3. The search strategy covers literature from the year 2000 to 2025. Given rapid changes in nutrition and health systems in many LMICs, data from 20-25 years ago may no longer accurately reflect current risk landscapes. How the authors clarify this?

Thank you for this pertinent observation. We acknowledge that the epidemiological and nutritional landscape in LMICs has shifted since 2000. To ensure our findings remain clinically

relevant, we will do a temporal sensitivity analysis. We proposed the following on line 299-303:

"To account for changes in the epidemiological and nutritional context in LMICs over time, we will conduct a temporal sensitivity analysis. Pooled risk factor estimates will be compared across two periods (2000–2012 and 2013–2025). This division reflects the introduction of the WHO's 2013 diagnostic criteria for gestational diabetes and the growing burden of maternal metabolic disorders in LMICs [50,51], both of which may influence the risk profile for LGA/macrosomia."

Comment 4. Although the protocol includes multiple standard definitions for LGA (90th vs. 97th percentile) and macrosomia (≥4000g vs. ≥4500g), the wide variety of thresholds used in primary studies can lead to significant clinical and statistical heterogeneity in meta-analyses.

Reponses: Thank you for this comment. To address this, we have proposed a specific subgroup analysis strategy (lines 291–298) to compare results based on the definitions used in the primary studies. Specifically, we will conduct separate analyses for: LGA: Comparing studies using the 90th percentile versus the 97th percentile; Macrosomia: Comparing studies using the ≥4000g threshold versus the ≥4500g threshold. By stratifying our meta-analysis by these definitions, we will be able to quantify the impact of different thresholds and ensure that the pooled estimates are methodologically sound and clinically interpretable.

"In subgroup analyses, we will compare common thresholds for LGA (90th vs. 97th percentile) and macrosomia (≥4000 g vs. ≥4500 g). We will also compare studies using INTERGROWTH versus other growth references and conduct stratified analyses by gestational age assessment method (ultrasound vs. LMP), where reported. While these stratifications help account for methodological differences, residual confounding may persist due to within-subgroup variability in gestational age assessment, growth references, and outcome definitions, as well as heterogeneity in measurement accuracy across settings. This variability may slightly affect the comparability of effect estimates and attributable fractions".

Comment 5. The decision to exclude meta-analysis for risk factors reported in fewer than three studies is statistically sound but may result in the omission of emerging or contextually important modifiable factors that are currently under-researched.

Reponses: Thank you for this comment. Risk factors reported in fewer than three studies will be excluded from the meta-analysis, but information on the various risk factors concerned will be reported in the systematic review section (on line 182-186): "*For risk factors that are reported in fewer than three studies from LMICs, meta-analyses calculations will not be conducted due to limited data and concerns over statistical reliability [38]. Instead, these factors will be described in systematic review to highlight their potential contribution to LGA or macrosomia and to identify areas where further research is warranted."*

Comment 6. The formula for adjusting the prevalence of gestational diabetes based on "ANC quality" (defined as the percentage of facilities willing to screen) is a creative approach, but it may oversimplify the actual clinical management and treatment adherence of pregnant women in practice.

Reponses: Thank you for this comment. We acknowledge that using ANC facility willingness as a proxy for quality of care may oversimplify real-world clinical management and patient adherence. We presented this as a limitation of the study (on line 344-348): "*While adjusting gestational diabetes prevalence according to the quality of antenatal care serves as a proxy for facility-level screening and management capacity, it does not capture individual-level factors such as follow-up or treatment adherence that may influence clinical outcomes. Consequently, some estimation bias may persist, as the detection and management of gestational diabetes can vary across settings*."

Comment 7. By only including studies that provide quantitative data on risk factors, the review may miss qualitative insights into the drivers of these risk factors (e.g., cultural beliefs regarding "eating for two" or barriers to physical activity). The review may benefit if it plans also to include these barriers in the analysis plan.

Reponses: Thank you for this comment. All studies reporting information on risk factors for large-for-gestational-age (LGA) or macrosomic births will be included in the systematic review. However, because the objective of this work is to identify risk factors for LGA and macrosomia in LMICs and quantify their population attributable fractions, only studies providing quantitative effect estimates will be included in the statistical pooling for the meta-analysis and PAF estimation. (line 154-157):

"*All studies reporting information on risk factors for LGA or macrosomia will be included in the systematic review. However, only studies providing quantitative data on the relationships between the prevalence or relationships between risk factors and LGA or macrosomia will be included in the meta-analysis.*"

Comment 8. The estimation of PAFs is limited only to countries included in the "Countdown to 2030" initiative. This excludes other LMICs that may also face a high burden of LGA or macrosomia but fall outside this specific global health group.

Reponses: Thank you for this comment. We have taken your suggestion into account and considered the list of countries proposed by the World Bank Group (on line 244-246): "*This process will be applied to all countries included in the list of LMICs proposed by the World Bank Group(34). The resulting estimates will enable us to quantify the absolute burden of LGA or macrosomia births by country.*"

Reviewer:1
If you have selected 'Yes' above, please provide details of any competing interests.: Not applicable

Reviewer:2
If you have selected 'Yes' above, please provide details of any competing interests.: Not applicable

VERSION 2 - REVIEW

**Reviewer**	**2**
**Name**	**Mengesha, Meresa Berwo**
**Affiliation**	**Adigrat University College of Health Sciences, Midwifery**
**Date**	**25-Mar-2026**
**COI**	

Congratulations, all the concerns raised were adequately addressed.
